# Explainable multi-modal radiomics for early prediction of liver metastasis in rectal cancer: a multicentric study

**DOI:** 10.1186/s13244-025-02010-9

**Published:** 2025-06-27

**Authors:** Yaru Feng, Jing Gong, Yanyan Wang, Yanfen Cui, Tong Tong

**Affiliations:** 1 Department of Radiology, Fudan University Shanghai Cancer Center and Department of Oncology, Shanghai Medical College, Fudan University, Shanghai, P. R. China; 2 Department of Radiology, Shanxi Province Cancer Hospital/Shanxi Hospital Affiliated to Cancer Hospital, Chinese Academy of Medical Sciences/Cancer Hospital Affiliated to Shanxi Medical University, Taiyuan, P. R. China

**Keywords:** Radiomics, Rectal cancer, Liver metastasis, Prediction

## Abstract

**Objectives:**

To enhance liver metastasis (LM) risk prediction for rectal cancer (RC) patients using a multi-modal, explainable radiomics model based on rectal MRI and whole-liver CT, and to assess its prognostic value for survival.

**Methods:**

This retrospective study enrolled patients with pathologically confirmed RC from two medical centers. Radiomics features were extracted from rectal MRI as well as pre-metastatic liver CT. Feature selection was performed using ANOVA F-value and recursive feature elimination. The SHAP method elucidated the model’s functionality by highlighting key feature contributions. Finally, Kaplan–Meier survival analysis and Cox regression assessed the prognostic utility of the model’s prediction score.

**Results:**

A total of 431 patients were enrolled from two centers in our study. The radiomics model developed from baseline whole-liver CT features alone could predict LM development in all cohorts. A fusion model integrating liver CT with primary tumor MRI features provided synergetic effect and was more efficient in predicting LM, displaying an area under the receiver operating curve (AUC) of 0.85 (95% CI: 0.80–0.90) in the training cohort, and AUC values of 0.75 (95% CI: 0.64–0.86) and 0.73 (95% CI: 0.61–0.85) in the internal and external validation cohorts, respectively. SHAP summary plots illustrated how feature values influenced their impact on the model. The risk score generated by our model demonstrated significant prognostic value for LM-free survival (LMFS).

**Conclusions:**

The multi-modal, explainable radiomics model integrating primary tumor and pre-metastatic liver radiomics enhances the prediction of LM development and provides prognostic value in RC patients.

**Critical relevance statement:**

This study demonstrates that integrating radiomics features from pre-metastatic liver and primary tumors enhances the predictive performance for liver metastasis development in rectal cancer patients, highlighting its potential for personalized treatment planning and follow-up strategies for rectal cancer patients.

**Key Points:**

- Pre-metastatic liver CT radiomics features could predict the liver metastasis development of rectal cancer.
- Integrating primary tumor and pre-metastatic liver radiomics improved liver metastasis prediction accuracy.
- The model demonstrated favorable interpretability through SHAP method.

**Graphical Abstract:**

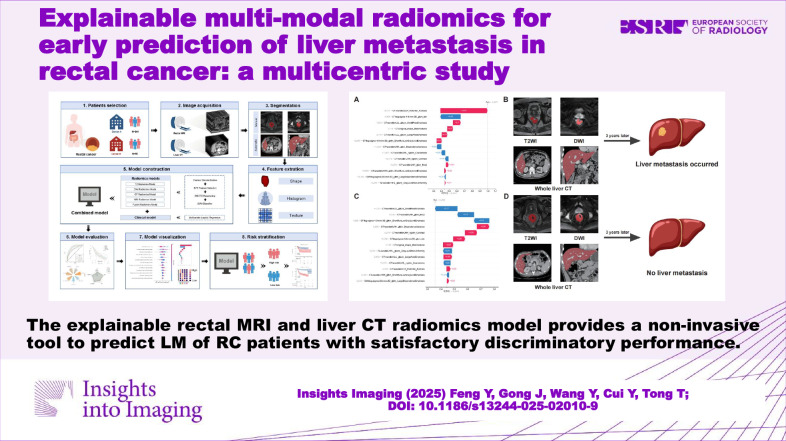

## Introduction

Rectal cancer (RC) is among the most common malignancies of the gastrointestinal tract globally, with the liver being the primary site of metastasis, associated with a lower survival rate [1]. At diagnosis, approximately 50–60% colorectal cancer patients have developed colorectal liver metastasis, with about 15–20% subsequently developing metachronous liver metastasis (LM) [2]. However, since current imaging technologies often detect tumors only when they are relatively large (> 1 cm³), about 90% of RC patients with LM do not receive an accurate early diagnosis, resulting in poor clinical outcomes [3, 4]. Thus, it is necessary to develop a predictive model to predict the LM risk of RC patients to identify potential patients who may benefit from liver resection.

Research indicates that high advanced T stages, lymph node metastasis, and male gender are risk factors for LM in RC [5–7], but their predictive accuracy is limited. Pelvic MRI remains the gold standard for local staging of RC; contrast-enhanced abdominal CT is the preferred modality for LM detection [8], while radiomics, a rapidly advancing field, extracts high-dimensional features that provide insights beyond traditional imaging [9–11], which could enhance our understanding of tumor biology and the cellular microenvironment.

Tumors establish a pre-metastatic niche (PMN) in target organs prior to metastasis, characterized by molecular and cellular alterations that create a favorable microenvironment—termed the “fertile soil”—to support metastatic colonization and promote distant organ metastasis. Based on this theory, it is hypothesized that in patients who eventually develop LM, PMN-associated microenvironmental changes in the liver occur prior to detection by radiological assessments [12, 13]. Current studies have attempted to predict LM by analyzing liver parenchyma CT radiomics [14–22]. However, existing studies using rectal CT or MRI radiomics for LM prediction in RC have primarily focused on the primary tumor microenvironment, neglecting the changes in the liver that occur prior to metastasis. Moreover, existing radiomic studies have yet to simultaneously explore both the tumor microenvironment and the pre-metastatic liver microenvironment, which limits the broader clinical applicability of radiomics.

To address these challenges, we develop and validate multi-modal radiomics model using rectal MRI and pre-metastatic whole-liver CT to predict LM in patient with RC. Additionally, we aim to explain our model to enhance the model’s explanation.

## Methods

### Study population

**Fig. 1:**
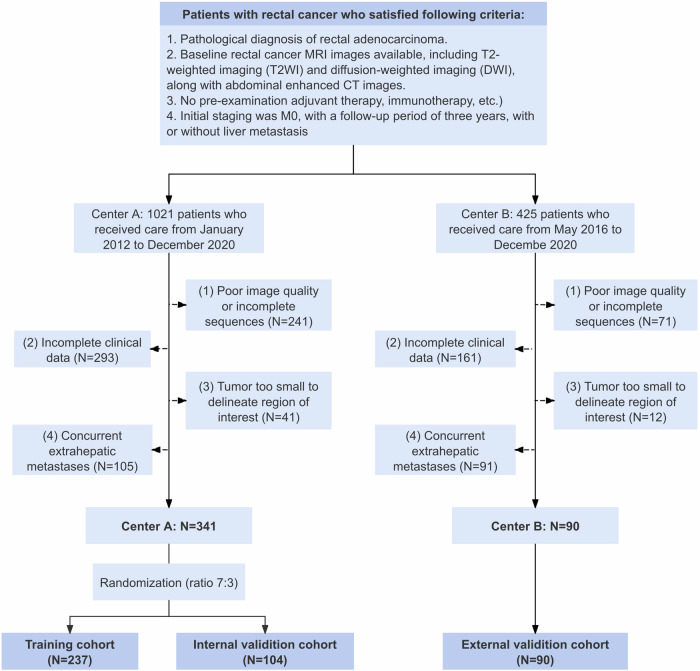
Flow chart of patient enrollment for each data set

This retrospective multicenter study adhered to the ethical principles outlined in the Declaration of Helsinki. The study was approved by the institutional ethics review boards, and the requirement for informed consent was waived due to its retrospective nature. We screened 431 patients with RC from Center A (Fudan University Shanghai Cancer Center) between January 2012 and December 2020, and from Center B (Shanxi Province Cancer Hospital) between May 2016 and December 2020. The specific inclusion and exclusion criteria are depicted in Fig. 1. Finally, patients from Center A were randomly assigned to the training and internal validation cohorts in a 7:3 ratio. Patients from Center B served as the independent external validation cohort. Clinical data acquisition methods and treatment strategies are documented in Appendices E1 and E2, while the diagnostic criteria for LM and follow-up protocols are outlined in Appendix E3.

### Image acquisition and segmentation

**Fig. 2:**
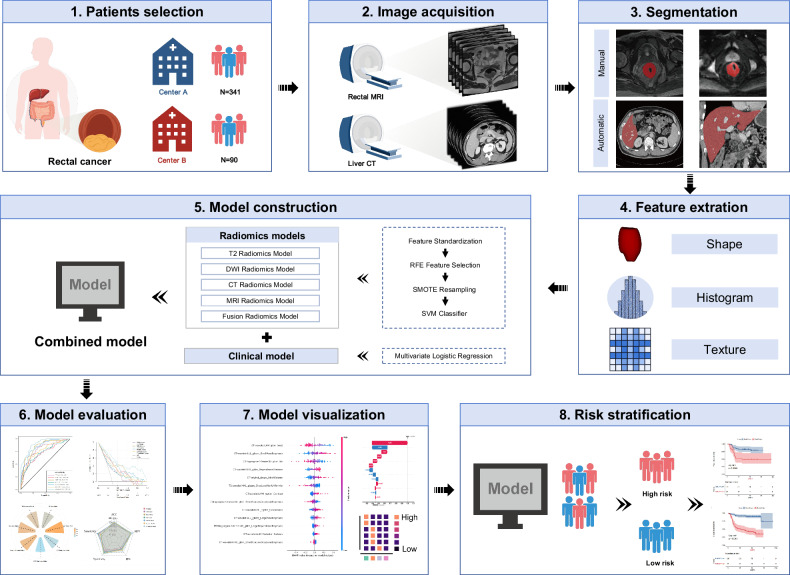
Overview of the overall research design. SVM, support vector machine; RFE, recursive feature elimination; SMOTE, synthetic minority oversampling technique

A schematic diagram of overall workflow is shown in Fig. 2. Rectal MRI and enhanced abdominal CT scans were performed within 2 weeks prior to treatment initiation, with a maximum interval of 1 week between examinations. The detailed MRI and CT scanning parameters are listed in Tables S1 and S2.

The rectal tumors were delineated by two junior radiologists (Y.F. and Y.W., with 4 years of experience) in a slice‑by‑slice manner. And the 3-dimensional (3D) volume of interest (VOI) from the whole-liver parenchyma without lesions was automatically segmented and subsequently manually adjusted by a junior radiologist (Y.F.). All segmentations were reviewed by two senior radiologists (T.T. and Y.C., with over 20 years of experience). The specific delineation details are provided in Appendix E4.

### Radiomics features extraction and selection

The radiomics features were extracted separately from the T2WI, DWI and CT using the PyRadiomics package (Version 2.1.2; https://pyradiomics.readthedocs.io). The details of the radiomics feature extraction are provided in Appendix E5. Finally, a total of 1046 radiomic features were extracted from T2WI and DWI based on the segmented primary rectal tumor, while 1106 radiomic features were extracted from the segmented pre-metastatic liver CT images.

### Model construction and evaluation

After extracting radiomic features of the primary tumor and the pre‑metastatic liver, a standard scaler was applied to standardize each imaging feature by removing the mean and scaling to unit variance. To reduce the dimensionality of MRI and CT image features, a two-step feature selection method was employed to select the optimal radiomics features. First, the top 20% of radiomic features with the highest ANOVA F-values were selected. Subsequently, recursive feature elimination (RFE) was used to remove redundant features. The RFE feature selection was configured with a linear support vector machine (SVM) estimator. Given the class imbalance between LM and non-LM patients in the training cohort, a synthetic minority oversampling technique (SMOTE) was applied to resample the cohort by increasing the number of minority cases. An SVM classifier was then used to build a predictive model for distinguishing between LM and non-LM patients. The MRI radiomics model and CT radiomics model were developed using the same feature processing techniques and SVM classifier. To further enhance model performance, a feature-level fusion strategy was applied, combining the prediction scores from the MRI and CT radiomics models. In this step, CT and MRI features were concatenated to create a fused feature pool. The same feature engineering techniques applied in the individual MRI and CT radiomics models, such as feature selection, SMOTE resampling, and SVM classification, were utilized to train and develop a fused feature model. The radiomics model development process are illustrated in Fig. 2.

Finally, we constructed seven models, including T2WI model, DWI model, the liver CT model, the rectal MRI (T2WI + DWI) model, the fusion model (MRI + CT), a clinical model, and a combined model (fusion radiomics features and clinical variables). The clinical variables associated with 3-year LM from the training cohort were selected through the least absolute shrinkage and selection operator (LASSO) regression analysis. And the clinical model was developed using features selected by LASSO regression, specifically those with non-zero regression coefficients. All models were constructed with the features of the training cohort and then applied to the internal validation and external validation cohorts.

To evaluate the performance of our proposed radiomics model, the area under the receiver operating characteristic (ROC) curve (AUC) and the corresponding 95% confidence interval (CI) were computed. The DeLong test was employed to compare the ROCs of different radiomic models. Moreover, several quantitative metrics, accuracy, sensitivity, specificity, positive predictive value, and negative predictive value, were calculated to assess the performance of our proposed radiomics model. The decision curve analysis (DCA) was performed to estimate the clinical usefulness of the models at different threshold probabilities.

We also investigated whether patients with different probabilities of LM predicted by the combined model could be stratified according to different risks of survival in these cohorts. Based on the optimum cutoff values identified by using the maximally selected rank statistical method of the combined model, the patients were categorized into low- and high-risk groups for LM.

### Model explanation and visualization

To interpret and understand the radiomic features from the radiomics model, we applied Shapley (SHAP) value, which is a game-theoretic approach to explain the output of a tree-based machine learning model [23]. The SHAP value plots can illustrate the positive and negative contributions of the features to the model (i.e., the probability of the prediction of LM in this study). Also, a heatmap was generated to visualize the distribution of variables for each patient across all cohorts. The combined model was visualized using a nomogram.

### Statistical analysis

Continuous variables with normal distribution are expressed as mean ± SD; otherwise, as the median and interquartile range (IQR). Categorical variables are expressed as numbers and percentages (*n*, %). The Wilcoxon signed rank or Kruskal–Wallis tests were used for numerical variables, and the χ^2^-test was used for categorical variables. The Kaplan–Meier method was used to generate survival curves, and the log-rank test was performed. Univariate and multivariate analyses were performed using the Cox proportional hazards model. All statistical tests were two-sided with a significance level of *p* < 0.05. Data were analyzed using R (Version 4.2.2, www.r-project.org).

## Results

### Clinical characteristics of patients

A total of 431 RC patients [median age, 59.0 (range, 50.0–65.0) years; 296 (66.8%) males] were initially acquired from two centers. The final training and internal validation cohorts consisted of 237 and 104 patients from Center A, and the external validation cohort included 90 patients from Center B. Table 1 provides a summary of the clinicopathological information of the patients across different cohorts. The most common clinical stage was stage III, with a frequency of 80.97, 78.9, and 81.73% in the training, internal validation, and external validation cohorts. Among the 431 patients, 122 developed LM within 3 years after baseline treatment, and occurred in 28.27, 28.85, and 27.78% of patients, respectively, with no inter-cohort difference (*p* = 0.986). The detailed clinical characteristics of the internal and external validation cohorts are shown in Table 2.

**Table 1:** Patient characteristics across different cohorts

Characteristics	Total (*n* = 431)	Internal training cohort (*n* = 237)	Internal validation cohort (*n* = 104)	External test cohort (*n* = 90)	*p*-value
Age (years)	59.00 (50.00–65.00)	58.00 (50.00–66.00)	60.50 (49.75–66.00)	58.00 (50.25–63.00)	0.684
Gender					0.091
Female	145 (33.64%)	74 (31.22%)	32 (30.77%)	39 (43.33%)	
Male	286 (66.36%)	163 (68.78%)	72 (69.23%)	51 (56.67%)	
CEA (ng/mL)					0.004
< 5	230 (53.36%)	115 (48.52%)	53 (50.96%)	62 (68.89%)	
≥ 5	201 (46.64%)	122 (51.48%)	51 (49.04%)	28 (31.11%)	
CA19_9 (μg/mL)					0.185
< 27	318 (73.78%)	172 (72.57%)	73 (70.19%)	73 (81.11%)	
≥ 27	113 (26.22%)	65 (27.43%)	31 (29.81%)	17 (18.89%)	
mrT stage					0.605
2	28 (6.50%)	14 (5.91%)	5 (4.81%)	9 (10.00%)	
3	296 (68.68%)	166 (70.04%)	71 (68.27%)	59 (65.56%)	
4	107 (24.83%)	57 (24.05%)	28 (26.92%)	22 (24.44%)	
mrN stage					< 0.001
0	82 (19.03%)	50 (21.10%)	19 (18.27%)	13 (14.44%)	
1	140 (32.48%)	64 (27.00%)	27 (25.96%)	49 (54.44%)	
2	209 (48.49%)	123 (51.90%)	58 (55.77%)	28 (31.11%)	
Distance (cm)	5.00 (3.60–7.15)	5.00 (3.70–7.00)	4.70 (3.27–6.78)	6.15 (3.82–8.00)	0.023
Length (cm)	4.90 (3.90–6.00)	5.00 (4.00–6.20)	4.90 (3.70–6.00)	4.35 (3.50–5.00)	< 0.001
MRF					0.037
Negative	198 (45.94%)	107 (45.15%)	40 (38.46%)	51 (56.67%)	
Positive	233 (54.06%)	130 (54.85%)	64 (61.54%)	39 (43.33%)	
mrTD					0.064
Negative	363 (84.22%)	195 (82.28%)	85 (81.73%)	83 (92.22%)	
Positive	68 (15.78%)	42 (17.72%)	19 (18.27%)	7 (7.78%)	
mrEMVI					0.526
Negative	202 (46.87%)	113 (47.68%)	44 (42.31%)	45 (50.00%)	
Positive	229 (53.13%)	124 (52.32%)	60 (57.69%)	45 (50.00%)	
Differentiation					< 0.001
Poorly differentiated	50 (11.60%)	34 (14.35%)	16 (15.38%)	0 (0.00%)	
Moderately differentiated	276 (64.04%)	141 (59.49%)	63 (60.58%)	72 (80.00%)	
Well-differentiated	25 (5.80%)	9 (3.80%)	5 (4.81%)	11 (12.22%)	
Absent	80 (18.56%)	53 (22.36%)	20 (19.23%)	7 (7.78%)	
Clinical stage					0.715
I	16 (3.71%)	9 (3.80%)	4 (3.85%)	3 (3.33%)	
II	66 (15.31%)	41 (17.30%)	15 (14.42%)	10 (11.11%)	
III	349 (80.97%)	187 (78.90%)	85 (81.73%)	77 (85.56%)	
KRAS					< 0.001
Negative	82 (19.03%)	35 (14.77%)	15 (14.42%)	32 (35.56%)	
Positive	108 (25.06%)	59 (24.89%)	26 (25.00%)	23 (25.56%)	
Absent	241 (55.92%)	143 (60.34%)	63 (60.58%)	35 (38.89%)	
NRAS					< 0.001
Negative	182 (42.23%)	92 (38.82%)	40 (38.46%)	50 (55.56%)	
Positive	8 (1.86%)	2 (0.84%)	1 (0.96%)	5 (5.56%)	
Absent	241 (55.92%)	143 (60.34%)	63 (60.58%)	35 (38.89%)	
BRAF					0.005
Negative	188 (43.62%)	93 (39.24%)	40 (38.46%)	55 (61.11%)	
Positive	2 (0.46%)	1 (0.42%)	1 (0.96%)	0 (0.00%)	
Absent	241 (55.92%)	143 (60.34%)	63 (60.58%)	35 (38.89%)	
Liver metastasis					0.986
No	309 (71.69%)	170 (71.73%)	74 (71.15%)	65 (72.22%)	
Yes	122 (28.31%)	67 (28.27%)	30 (28.85%)	25 (27.78%)	
Treatment					< 0.001
Surgery after NCRT	192 (44.55%)	140 (59.07%)	52 (50.00%)	0 (0.00%)	
Only surgery	169 (39.21%)	51 (21.52%)	28 (26.92%)	90 (100.00%)	
Only NCRT	70 (16.24%)	46 (19.41%)	24 (23.08%)	0 (0.00%)	

*CA19_9* carbohydrate antigen 19_9, *CEA* carcinoembryonic antigen, *EMVI* extramural venous invasion, *MRF* mesorectal fascia, *TD* tumor deposits, *NCRT* neoadjuvant chemoradiotherapy

**Table 2:** Comparison of clinical and pathological characteristics between the LM group and the non-LM group

Characteristics	Internal training cohort (*n* = 237)		*p*-value	Internal validation cohort (*n* = 104)		*p*-value	External test cohort (*n* = 90)		*p*-value
	Non-LM (*n* = 170)	LM (*n* = 67)		Non-LM (*n* = 74)	LM (*n* = 30)		Non-LM (*n* = 65)	LM (*n* = 25)	
Age (years)	58.50 (51.00–66.00)	57.00 (48.50–64.50)	0.550	57.50 (47.00–65.00)	65.00 (57.75–70.50)	0.002	56.66 ± 8.43	59.80 ± 9.99	0.137
Distance (cm)	4.65 (3.60–6.65)	5.50 (4.00–8.20)	0.021	4.50 (3.20–6.20)	5.00 (3.78–7.30)	0.283	6.00 (3.70–8.00)	6.50 (5.00–8.00)	0.288
Length (cm)	5.00 (4.00–6.68)	4.90 (4.05–6.00)	0.296	5.00 (3.82–6.50)	4.55 (3.62–5.25)	0.246	4.23 ± 1.24	4.35 ± 1.20	0.676
Gender			0.980			0.130			0.022
Female	53 (31.18%)	21 (31.34%)		26 (35.14%)	6 (20.00%)		33 (50.77%)	6 (24.00%)	
Male	117 (68.82%)	46 (68.66%)		48 (64.86%)	24 (80.00%)		32 (49.23%)	19 (76.00%)	
CEA(ng/mL)			0.006			0.006			0.910
< 5	92 (54.12%)	23 (34.33%)		44 (59.46%)	9 (30.00%)		45 (69.23%)	17 (68.00%)	
≥ 5	78 (45.88%)	44 (65.67%)		30 (40.54%)	21 (70.00%)		20 (30.77%)	8 (32.00%)	
CA19_9 (μg/mL)			0.840			0.148			0.867
< 27	124 (72.94%)	48 (71.64%)		55 (74.32%)	18 (60.00%)		53 (81.54%)	20 (80.00%)	
≥ 27	46 (27.06%)	19 (28.36%)		19 (25.68%)	12 (40.00%)		12 (18.46%)	5 (20.00%)	
mrT stage			0.078			0.163			0.076
2	12 (7.06%)	2 (2.99%)		5 (6.76%)	0 (0.00%)		8 (12.31%)	1 (4.00%)	
3	112 (65.88%)	54 (80.60%)		47 (63.51%)	24 (80.00%)		45 (69.23%)	14 (56.00%)	
4	46 (27.06%)	11 (16.42%)		22 (29.73%)	6 (20.00%)		12 (18.46%)	10 (40.00%)	
mrN stage			0.088			0.659			< 0.001
0	42 (24.71%)	8 (11.94%)		15 (20.27%)	4 (13.33%)		12 (18.46%)	1 (4.00%)	
1	45 (26.47%)	19 (28.36%)		18 (24.32%)	9 (30.00%)		42 (64.62%)	7 (28.00%)	
2	83 (48.82%)	40 (59.70%)		41 (55.41%)	17 (56.67%)		11 (16.92%)	17 (68.00%)	
Distance (cm)	4.65 (3.60–6.65)	5.50 (4.00–8.20)	0.021	4.50 (3.20–6.20)	5.00 (3.78–7.30)	0.283	6.00 (3.70–8.00)	6.50 (5.00–8.00)	0.288
Length (cm)	5.00 (4.00–6.68)	4.90 (4.05–6.00)	0.296	5.00 (3.82–6.50)	4.55 (3.62–5.25)	0.246	4.23 ± 1.24	4.35 ± 1.20	0.676
MRF			0.096			0.259			0.014
Negative	71 (41.76%)	36 (53.73%)		31 (41.89%)	9 (30.00%)		42 (64.62%)	9 (36.00%)	
Positive	99 (58.24%)	31 (46.27%)		43 (58.11%)	21 (70.00%)		23 (35.38%)	16 (64.00%)	
mrTD			< 0.001			0.158			0.007
Negative	151 (88.82%)	44 (65.67%)		63 (85.14%)	22 (73.33%)		63 (96.92%)	20 (80.00%)	
Positive	19 (11.18%)	23 (34.33%)		11 (14.86%)	8 (26.67%)		2 (3.08%)	5 (20.00%)	
mrEMVI			0.153			0.893			0.002
Negative	86 (50.59%)	27 (40.30%)		31 (41.89%)	13 (43.33%)		39 (60.00%)	6 (24.00%)	
Positive	84 (49.41%)	40 (59.70%)		43 (58.11%)	17 (56.67%)		26 (40.00%)	19 (76.00%)	
Differentiation			0.055			0.203			-
Poorly differentiated	28 (16.47%)	6 (8.96%)		10 (13.51%)	6 (20.00%)		0 (0.00%)	0 (0.00%)	
Moderately differentiated	92 (54.12%)	49 (73.13%)		43 (58.11%)	20 (66.67%)		52 (80.00%)	20 (80.00%)	
Well-differentiated	8 (4.71%)	1 (1.49%)		3 (4.05%)	2 (6.67%)		6 (9.23%)	5 (20.00%)	
Absent	42 (24.71%)	11 (16.42%)		18 (24.32%)	2 (6.67%)		7 (10.77%)	0 (0.00%)	
Clinical stage			0.086			0.410			0.115
I	7 (4.12%)	2 (2.99%)		4 (5.41%)	0 (0.00%)		2 (3.08%)	1 (4.00%)	
II	35 (20.59%)	6 (8.96%)		11 (14.86%)	4 (13.33%)		10 (15.38%)	0 (0.00%)	
III	128 (75.29%)	59 (88.06%)		59 (79.73%)	26 (86.67%)		53 (81.54%)	24 (96.00%)	
KRAS			< 0.001			0.129			0.117
Negative	18 (10.59%)	17 (25.37%)		8 (10.81%)	7 (23.33%)		19 (29.23%)	13 (52.00%)	
Positive	35 (20.59%)	24 (35.82%)		17 (22.97%)	9 (30.00%)		19 (29.23%)	4 (16.00%)	
Absent	117 (68.82%)	26 (38.81%)		49 (66.22%)	14 (46.67%)		27 (41.54%)	8 (32.00%)	
NRAS			< 0.001			0.124			0.632
Negative	53 (31.18%)	39 (58.21%)		24 (32.43%)	16 (53.33%)		35 (53.85%)	15 (60.00%)	
Positive	0 (0.00%)	2 (2.99%)		1 (1.35%)	0 (0.00%)		3 (4.62%)	2 (8.00%)	
Absent	117 (68.82%)	26 (38.81%)		49 (66.22%)	14 (46.67%)		27 (41.54%)	8 (32.00%)	
BRAF			< 0.001			0.072			-
Negative	53 (31.18%)	40 (59.70%)		25 (33.78%)	15 (50.00%)		38 (58.46%)	17 (68.00%)	
Positive	0 (0.00%)	1 (1.49%)		0 (0.00%)	1 (3.33%)		0 (0.00%)	0 (0.00%)	
Absent	117 (68.82%)	26 (38.81%)		49 (66.22%)	14 (46.67%)		27 (41.54%)	8 (32.00%)	
Treatment			0.051			0.021			-
Surgery after NCRT	99 (58.24%)	41 (61.19%)		36 (48.65%)	16 (53.33%)		0 (0.00%)	0 (0.00%)	
Only surgery	32 (18.82%)	19 (28.36%)		16 (21.62%)	12 (40.00%)		65 (100.00%)	25 (100.00%)	
Only NCRT	39 (22.94%)	7 (10.45%)		22 (29.73%)	2 (6.67%)		0 (0.00%)	0 (0.00%)	

*CA19_9* carbohydrate antigen 19_9, *CEA* carcinoembryonic antigen, *EMVI* extramural venous invasion, *MRF* mesorectal fascia, *TD* tumor deposits, *NCRT* neoadjuvant chemoradiotherapy, *LM* liver metastasis

### Clinical model performance

Through LASSO regression analysis from the training cohort, preliminary screening of clinical variables related to 3-year LM status was conducted. Finally, nine clinical variables, including age, CEA, CA19_9, distance from the anus, length, N stage, MRF, mrTD, and mrEMVI, were determined to construct the clinical model to predict 3-year LM (Fig. S1).

**Fig. 3:**
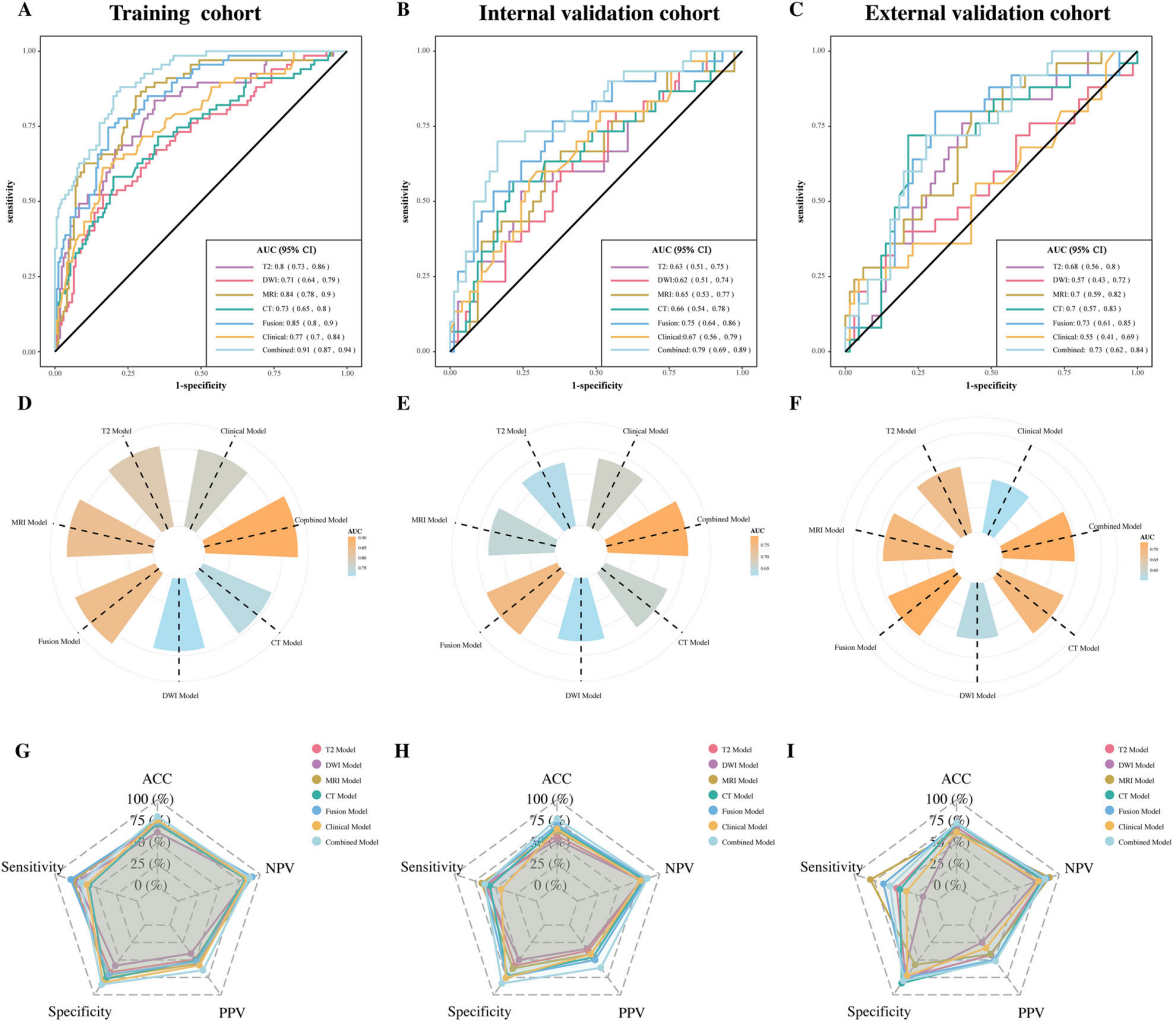
Performance of the seven models for predicting liver metastasis within 3 years, including T2WI model, DWI model, the liver CT model, the rectal MRI (T2WI + DWI) model, the fusion model (MRI + CT), a clinical model, and a combined model (fusion radiomics features and clinical variables). **A**–**C** Display of receiver operating characteristic curves across training, internal validation, and external validation cohorts. Area under the receiver operating characteristic (AUC) of the seven models in training, internal validation, and external validation cohorts. **D**–**F** Color-coded circular barplots visually depict the AUC values for each model in training, internal, and external validation cohorts. **G**–**I** Radar plots illustrating the comparative discrimination performance of the models across training, internal, and external validation cohorts. ACC, accuracy; AUC, area under the curve; CI, confidence interval; PPV, positive predictive value; NPV, negative predictive value

The training cohort showed an AUC of 0.77 (95% CI, 0.7–0.84). This was later verified with AUCs of 0.67 (95% CI, 0.56–0.79) in the internal validation cohort and 0.55 (95% CI, 0.41–0.69) in the external validation cohort, respectively (Fig. 3).

### Radiomics signature construction

Figure S2 illustrates the boxplots of the selected radiomic features in the T2WI model, DWI model, the liver CT model, the rectal MRI (T2WI + DWI) model and the fusion feature model representing the rectal MRI and abdomen CT radiomic features, with a total of 14, 6, 6, 16, and 13 radiomic features were selected respectively. The specific features were listed in Table S3.

### Prediction performance of the radiomics-based model

Table 3 and Fig. 3 comprehensively summarize the performance metrics of all models across training, internal, and external validation cohorts. Both LM prediction models developed from the MRI and CT radiomic features showed acceptable predictive efficacy. The MRI-based radiomics model demonstrated AUCs of 0.84 (95% CI: 0.78–0.90), 0.65 (0.53–0.77), and 0.70 (0.59–0.82) in the training, internal validation, and external validation cohorts, respectively. The CT-based model achieved AUCs of 0.73 (0.65–0.80), 0.66 (0.54–0.78), and 0.70 (0.57–0.83) across corresponding cohorts. Comparatively, the combined model demonstrated superior discriminative capacity with AUCs of 0.91 (95% CI: 0.87–0.94), 0.79 (0.69–0.89), and 0.73 (0.62–0.84) in the training, internal validation, and external validation cohorts, respectively. DeLong tests confirmed the combined model’s superior performance over both MRI- and CT-based radiomics models (all *p* < 0.05) in training and internal validation cohorts (Table S4).

**Table 3:** Prediction performance of all models

Models	Datasets	AUC	95% CI	ACC %	Sensitivity	Specificity	PPV	NPV
DWI radiomics model	Training cohort	0.71	[0.64, 0.77]	62.45	0.7313	0.5824	0.4083	0.8462
	Internal validation cohort	0.62	[0.53, 0.73]	53.85	0.6333	0.5	0.3393	0.7708
	External validation cohort	0.57	[0.44, 0.69]	63.33	0.16	0.8154	0.25	0.7162
T2 radiomics model	Training cohort	0.8	[0.74, 0.85]	70.89	0.791	0.6765	0.4907	0.8915
	Internal validation cohort	0.63	[0.53, 0.74]	58.65	0.6	0.5811	0.3673	0.7818
	External validation cohort	0.68	[0.57, 0.77]	67.78	0.48	0.7538	0.4286	0.7903
MRI radiomics model	Training cohort	0.84	[0.79, 0.88]	75.95	0.7761	0.7529	0.5532	0.8951
	Internal validation cohort	0.65	[0.55, 0.76]	63.46	0.6667	0.6216	0.4167	0.8214
	External validation cohort	0.7	[0.60, 0.80]	63.33	0.8	0.5692	0.4167	0.881
CT radiomics model	Training cohort	0.73	[0.66, 0.79]	72.15	0.5821	0.7765	0.5065	0.825
	Internal validation cohort	0.66	[0.55, 0.76]	68.27	0.5667	0.7297	0.4595	0.806
	External validation cohort	0.7	[0.58, 0.81]	72.22	0.44	0.8308	0.5	0.7941
Fusion radiomics model	Training cohort	0.85	[0.81, 0.89]	73.84	0.806	0.7118	0.5243	0.903
	Internal validation cohort	0.75	[0.65, 0.83]	71.15	0.6333	0.7432	0.5	0.8333
	External validation cohort	0.73	[0.62, 0.82]	72.22	0.64	0.7538	0.5	0.8448
Clinical model	Training cohort	0.77	[0.7, 0.84]	76.37	0.612	0.8235	0.577	0.843
	Internal validation cohort	0.67	[0.56, 0.79]	66.3	0.433	0.757	0.419	0.767
	External validation cohort	0.55	[0.41, 0.69]	62.2	0.36	0.723	0.33	0.746
Combined model	Training cohort	0.91	[0.87, 0.94]	80.5	0.686	0.852	0.647	0.873
	Internal validation cohort	0.79	[0.69, 0.89]	77.9	0.63	0.837	0.612	0.849
	External validation cohort	0.73	[0.62, 0.84]	73.3	0.56	0.8	0.518	0.826

*ACC* accuracy, *AUC* area under the curve, *CI* confidence interval, *PPV* positive predictive value, *NPV* negative predictive value

### Performance of the combined model for LM prediction

The combined model was constructed with the clinical variables selected by LASSO regression and fusion radiomics signature generated by MRI and CT. The AUC of the combined model for the prediction of LM was 0.91 (95% CI, 0.87–0.94; accuracy 80.5%; sensitivity 68.6%; specificity 85.2%) in the training cohort, 0.79 (95% CI, 0.69–0.89; accuracy 77.9%; sensitivity 63.0%; specificity 83.7%) in the internal validation cohort, and 0.73 (95% CI, 0.62–0.84; accuracy 73.3%; sensitivity 56.0%; specificity 80.0%) in the external validation cohort (Fig. 3, Table 3). The DCA revealed that the combined model provided a higher net benefit within a certain threshold range (Fig. S3). To enhance clinical applicability, the combined radiomics model was visualized using a nomogram (Fig. S4), with each variable’s contribution quantified by point scales.

### Model explanation with SHAP

The SHAP analysis revealed “glcm_Imc2” as the most discriminative feature for LM/non-LM classification in the fusion radiomics model (Fig. S5), with decreasing feature values corresponding to higher model output probabilities.

**Fig. 4:**
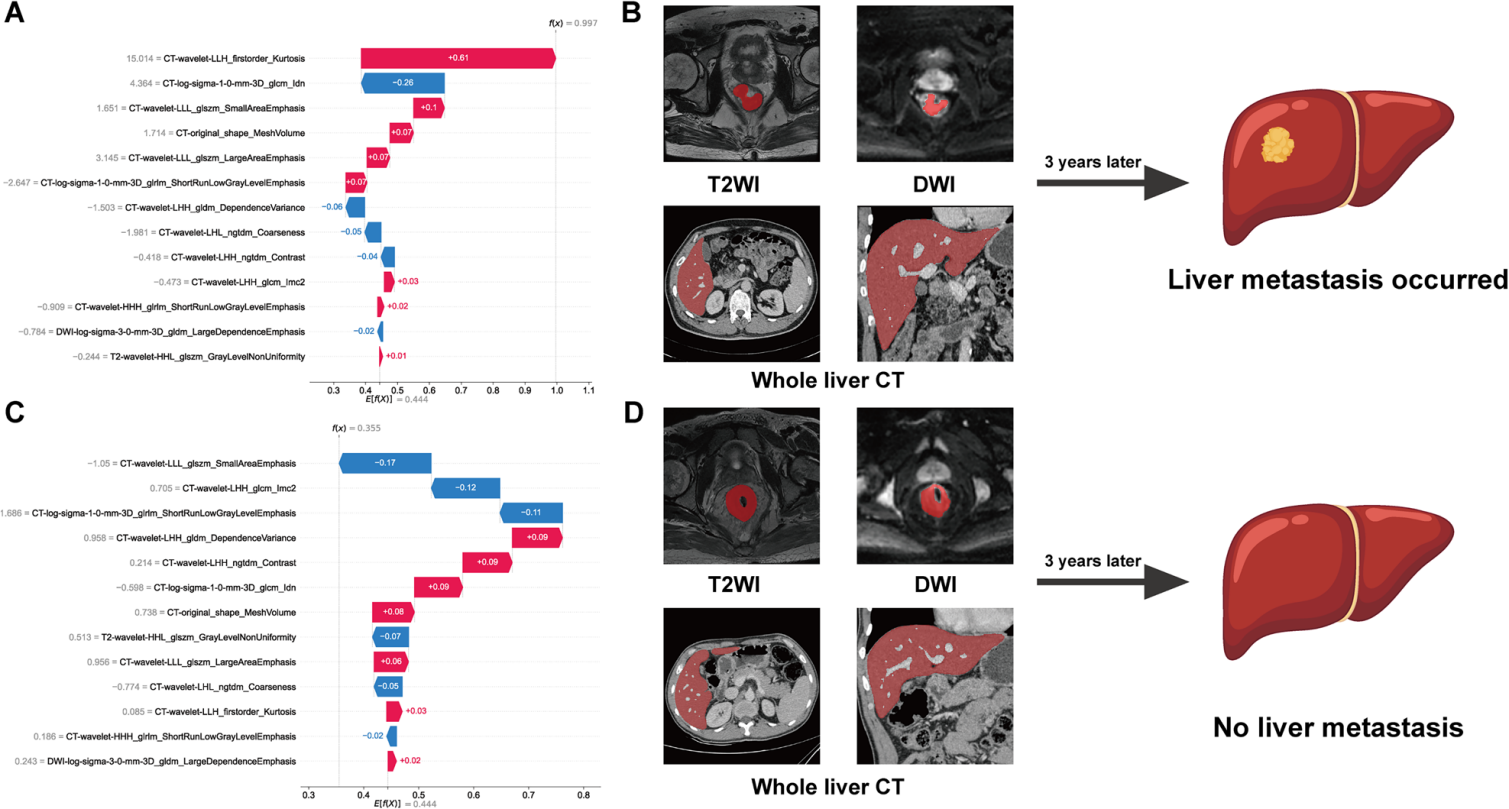
This figure illustrates how the fusion radiomics model differentiates between two patients in terms of predicting liver metastasis within 3 years. **A** The SHAP force plot for Patient A, classified into the LM group, where a high feature value (CT-wavelet-LLH first-order Kurtosis) contributed to the increased likelihood of liver metastasis classification. **B** Corresponding image segmentation examples for Patient A, including rectal T2WI, DWI, and pre-metastatic CT scans. **C** The SHAP force plot for Patient B, classified into the non-liver metastasis group, where a low feature value (CT-wavelet-LLL glszm_SmallAreaEmphasis) contributed to the assessment. **D** Corresponding image segmentation examples for Patient B, including rectal T2WI, DWI, and pre-metastatic CT scans

SHAP waterfall plots of two representative cases from the fusion model training cohort (Fig. 4) illustrated the classification mechanism. In the LM case (Fig. 4A, B), the SHAP value (0.997) exceeded the baseline (0.444), primarily driven by “firstorder_Kurtosis” (0.61). Conversely, the non-LM case (Fig. 4C, D) showed a lower SHAP value (0.355 vs. 0.444 baseline), with “glszm_SmallAreaEmphasis” (0.17) reducing metastasis likelihood.

### Prognostic value of the radiomics-based model

**Fig. 5:**
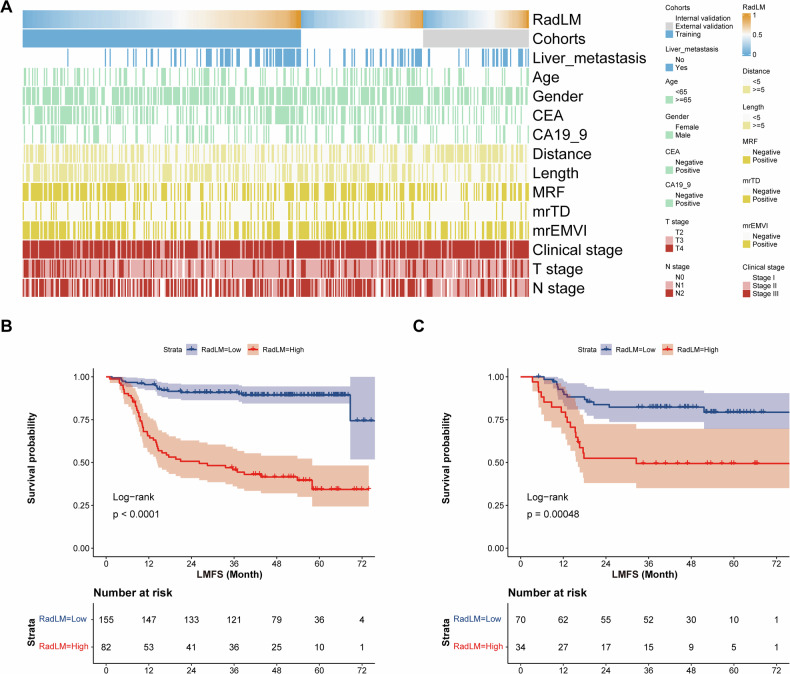
**A** A heatmap displays the distribution of clinical, MRI, and pathological features across patients in training, internal validation, and external validation cohorts. Each row in the heatmap represents a distinct factor, with colors defined in the legends to the right and above. **B**, **C** Kaplan–Meier curves show liver metastasis-free survival based on RadLM scores in the training and internal validation cohorts. Group comparisons were performed using the Log-rank test

The RadLM score was developed from the fusion radiomics model, with patient clinicopathological characteristics co-visualized in the heatmap (Fig. 5A). Using the optimal cutoff value (0.549) derived from the training cohort, patients were stratified into high- and low-risk subgroups. Kaplan–Meier analysis revealed significantly shorter liver metastasis-free survival (LMFS) in the high-risk subgroup compared to the low-risk group, consistent across both training and internal validation cohorts (Fig. 5B, C). Multivariable Cox regression analysis established the RadLM score as an independent prognostic factor after adjusting for potential confounding factors, with detailed results provided in Table 4. Subsequent subgroup analyses of surgical patients further validated the RadLM score’s predictive capacity for metastasis risk, demonstrating its prognostic independence from postoperative chemotherapy effects as documented in Table S5.

**Table 4:** Cox regression analysis for LM prediction in Center A

Variables	Classification	Numbers (proportion)	HR (univariable)	HR (multivariable)
Age (years)	< 60	176 (51.6%)		
	≥ 60	165 (48.4%)	1.46 (0.98–2.19, *p* = 0.061)	1.75 (1.14–2.67, *p* = 0.010)
Gender	Female	106 (31.1%)		
	Male	235 (68.9%)	1.19 (0.76–1.86, *p* = 0.438)	
CEA (ng/mL)	< 5	168 (49.3%)		
	≥ 5	173 (50.7%)	2.28 (1.49–3.50, *p* < 0.001)	2.21 (1.43–3.40, *p* < 0.001)
CA19_9 (μg/mL)	< 27	245 (71.8%)		
	≥ 27	96 (28.2%)	1.30 (0.85–2.00, *p* = 0.227)	
MRF	Negative	147 (43.1%)		
	Positive	194 (56.9%)	0.94 (0.63–1.40, *p* = 0.755)	
Distance (cm)	Low	203 (59.5%)		
	Middle or high	138 (40.5%)	0.58 (0.38–0.91, *p* = 0.017)	0.48 (0.30–0.75, *p* = 0.002)
Length (cm)	< 5	162 (47.5%)		
	≥ 5	179 (52.5%)	0.75 (0.50–1.12, *p* = 0.155)	
mrTD	Negative	280 (82.1%)		
	Positive	61 (17.9%)	2.83 (1.84–4.35, *p* < 0.001)	3.48 (2.22–5.46, *p* < 0.001)
mrEMVI	Negative	157 (46.0%)		
	Positive	184 (54.0%)	1.38 (0.92–2.08, *p* = 0.118)	
Clinical stage	I	13 (3.8%)		
	II	56 (16.4%)	1.19 (0.26–5.42, *p* = 0.825)	
	III	272 (79.8%)	2.30 (0.56–9.34, *p* = 0.245)	
RadLM score	Low-risk	225 (66.0%)		
	High-risk	116 (34.0%)	5.79 (3.76–8.92, *p* < 0.001)	5.50 (3.48–8.68, *p* < 0.001)
Treatment	Surgery after NCRT	192 (56.3%)		
	Only surgery	79 (23.2%)	1.43 (0.92–2.21, *p* = 0.111)	1.32 (0.83–2.10, *p* = 0.237)
	Only NCRT	70 (20.5%)	0.41 (0.21–0.84, *p* = 0.014)	0.60 (0.29–1.23, *p* = 0.162)

*CA19_9* carbohydrate antigen 19_9, *CEA* carcinogenicity antigen, *EMVI* extramural venous invasion, *MRF* mesorectal fascia, *TD* tumor deposits, *NCRT* advantageous chemoradiotherapy, *HR* hazard ratio

## Discussion

In this study, we developed an explainable multi-modal radiomics model to enhance LM prediction accuracy in RC patients. By integrating radiomics features from both primary tumors and pre-metastatic liver regions, the model showed superior predictive performance for LM development. SHAP analysis improved interpretability by quantifying feature contributions. The model also effectively stratifies RC patients into high- and low-risk groups, emphasizing the prognostic value of our multi-modal approach.

To the best of our knowledge, this is the first study to integrate rectal MRI and pre-metastatic liver CT radiomics features to predict LM development in RC patients, emphasizing the importance of multi-modal and multi-organ fusion radiomics analysis. Previous studies predominantly using single-modality imaging (primary tumor or liver radiomics features) showed inadequate characterization of tumor microenvironment heterogeneity, particularly limited in predicting distant metastasis [18, 19, 21, 22]. Additionally, these studies typically lacked external validation, or their model performance was inferior to that of our study. Consequently, our integrated model offers advantages in clinical applicability compared to traditional unimodal approaches. By integrating radiomic features from both primary tumors and the pre-metastatic liver, this approach reveals tumor microenvironment characteristics and evaluates metastatic proliferative potential in the target organ. Similarly, a study on lung cancer brain metastasis demonstrated that combining features from primary and pre-metastatic organs provides insights into metastatic potential [24]. Overall, integrating rectal tumors and the pre-metastatic liver radiomic features enhances LM prediction accuracy through complementary information.

Furthermore, our study provides compelling evidence of pre-metastatic microenvironmental changes in the liver. Theoretically, primary tumors actively and specifically induce the formation of PMN, preparing the distant organ microenvironments for the colonization of disseminated tumor cells [25]. The radiomic features of the liver reflect its tissue heterogeneity, which may indicate the presence of micro or occult metastases. Recent studies suggest that imaging techniques can non-invasively monitor changes in the PMNs, potentially aiding in more accurate identification of high-risk cancer patients prone to distant metastasis [25–27]. Our findings support this theory, as the inclusion of CT radiomic features in our fused model significantly enhanced its predictive performance compared to models based solely on primary tumors (Fig. 3). Additionally, our liver image analysis advances beyond prior studies. While existing hepatic parenchyma research predominantly used basic statistical methods on intensity features without identifying LM-related signatures [14–17, 20], our fused model incorporates 13 LM-associated radiomic features, surpassing previous clinical relevance. Lee et al’s single-slice approach [28] limited comprehensive liver evaluation, whereas our automated segmentation with minimal manual intervention ensures accurate, reproducible whole-organ feature capture. This pre-metastatic microenvironment-focused strategy could improve predictive performance in studies of other cancers or target organs.

We applied SHAP to clarify feature impacts in our fusion radiomics model to improve interpretability. Prior studies often lack interpretability, limiting clinical use. Among the features, “glcm_Imc2” from wavelet-transformed images had the most significant impact on the model’s decisions, as it quantifies texture complexity by assessing the correlation between two gray level distributions [11]. Previous studies suggest that this feature effectively distinguishes between the advancing and infiltrating fronts of tumor growth, thereby potentially revealing changes in the liver’s microenvironment before metastasis [29]. Based on these findings, we hypothesize that “glcm_Imc2” could serve as a predictive biomarker for LM. Additionally, the nomogram provides a visual representation of the combined model, enabling clinicians to intuitively interpret predictive results. Incorporating established risk determinants such as advanced age, low rectal tumor location, and elevated RadLM scores, the model identified patients with increased metastatic potential.

Finally, we evaluated the prognostic significance of the prediction scores generated by the fusion radiomics model in predicting LMFS. We hypothesized that these scores could also serve as predictors of LMFS, considering that the development of LM is a critical factor in the prognosis of RC. The analysis revealed that the model’s risk stratification independently predicts LMFS, even after adjusting for clinicopathological variables. Therefore, the integrated multi-modal radiomics model enables early identification of high-risk patients, suggesting that these patients may require intensified follow-up schedules and earlier therapeutic interventions.

Despite these findings, this study has several limitations. First, despite external validation, this retrospective study may introduce selection bias and hidden confounders. Second, despite utilizing image resampling techniques to standardize CT and MRI images, the variability inherent in different imaging parameters remains a significant challenge. Third, this study exclusively extracted radiomic features from T2WI and DWI MRI sequences and portal venous phase CT scans, omitting other imaging phases or sequences. Future studies should encompass a wider range of imaging phases and sequences. Lastly, due to the limited availability of survival data among patients, overall survival was not investigated. Future studies should focus on more prognostic outcome indicators.

In conclusion, we developed and validated a multi-modal radiomics model that enhances the ability to predict LM development in RC patients by integrating radiomics features from pre-metastatic liver and primary tumors. Additionally, we utilized SHAP values to explain our model. Therefore, this multi-modal, explainable model has the potential to assist clinicians in assessing LM risk, thereby facilitating personalized treatment planning and follow-up strategies for RC patients.

## Supplementary information

ELECTRONIC SUPPLEMENTARY MATERIAL

## Data Availability

Owing to patient privacy concerns, patients’ data are not publicly accessible. However, they are available upon reasonable request from the corresponding author (t983352@126.com).

## Abbreviations

AUC: Area under the curve
CA19_9: Carbohydrate antigen 19_9
CEA: Carcinoembryonic antigen
CI: Confidence interval
DCA: Decision curve analysis
HR: Hazard ratio
LM: Liver metastasis
MRF: Mesorectal fascia
NCRT: Neoadjuvant chemoradiotherapy
PMN: Pre-metastatic niche
RC: Rectal cancer
ROC: Receiver operating characteristic
SHAP: SHapley additive exPlanations
SVM: Support vector machine
