# Supplementary material for: Explainable multi-modal radiomics for early prediction of liver metastasis in rectal cancer: a multicentric study
Source: Insights Imaging. 2025 Jun 27;16:142. doi: 10.1186/s13244-025-02010-9 (PMC12205110; doi:10.1186/s13244-025-02010-9)
Supplement: Supplementary file 1 — ELECTRONIC SUPPLEMENTARY MATERIAL [file 13244_2025_2010_MOESM1_ESM.pdf]

# Explainable multi-modal radiomics for early prediction of liver metastasis in rectal cancer: A multicentric study

# ELECTRONIC SUPPLEMENTARY MATERIAL

**Appendix E1. Clinical data collection**

Detailed demographic, baseline clinical data were collected, including age, gender, carcinoembryonic antigen (CEA), carbohydrate antigen 19_9 (CA19_9), as well as the treatment plans. Additionally, MRI features such as T stage, N stage, distance from the anus, tumor length, extramural vascular invasion (EMVI), and mesorectal fascia (MRF) involvement were obtained from the electronic medical records. Pathological data, including tumor differentiation and gene mutations (KRAS, BRAF, and NARS), were retrieved from the pathology reports.

**Appendix E2. Treatment strategies**

**Center A:** The primary treatment strategies comprised three approaches, including surgery following neoadjuvant chemoradiotherapy (NCRT), surgery alone, and NCRT alone. The surgical procedure referred to total mesorectal excision (TME). For patients receiving NCRT, TME was performed within 6-8 weeks after completing NCRT.

**Center B:** The surgical procedure referred to TME.

**NCRT regimens:** Patients received radiotherapy using intensity-modulated radiation therapy (IMRT), with a total dose of 45-50 Gy administered in 25 fractions of 1.8-2 Gy each over 5 weeks (once daily on weekdays). Chemotherapy regimens included Capecitabine (625 mg/m$^2$, twice daily on Days 1-5 weekly) combined with either Irinotecan (65-80 mg/m$^2$, once weekly), Oxaliplatin (50 mg/m$^2$, once weekly), or as Capecitabine alone (825 mg/m$^2$, twice daily).

**Appendix E3. Diagnostic criteria for LM and follow-up protocol**

Metachronous liver metastasis (LM) was diagnosed according to NCCN (National Comprehensive Cancer Network) and ESMO (European Society for Medical Oncology) criteria[1,2], primarily relying on contrast-enhanced abdominal CT imaging. All enrolled patients had confirmed absence of LM at baseline imaging. During follow-up evaluations, newly identified suspicious hepatic lesions were systematically assessed: definitive diagnoses were established via contrast-enhanced CT for typical cases, while equivocal lesions underwent further characterization using liver MRI or PET/CT. Histopathological confirmation via puncture biopsy or surgical resection was required for imaging-indeterminate lesions.

Patients were followed every 3 months during the first year after treatment completion and every 6 months thereafter, with a minimum follow-up of 3 years (final follow-up date: December 31, 2023). Liver metastasis-free survival (LMFS) was defined as the interval from treatment completion to the first radiologically or pathologically confirmed LM, all-cause death, or the last documented disease-free follow-up.

**Appendix E4. Details of image segmentation**

For the primary tumor, ITK-SNAP software (Version: 3.4.3, www.itksnap.org) was used to manually delineate the region of interest (ROI) along the tumor border on each successive slice of the entire tumor using T2-weighted imaging (T2WI) and diffusion-weighted imaging (DWI) (with a b-value of 800 s/mm$^2$). The ROI was outlined for the entire tumor volume on each slice, excluding surrounding lymph nodes. Cystic and necrotic areas were manually excluded from the segmentation.

For liver segmentation, all selected patients at both institutions underwent contrast-enhanced abdominal CT using 64-row spiral CT scanners. The entire liver was segmented from portal venous phase (PVP) contrast-enhanced CT images using the imaging platform uAI Research Portal software (uRP) (United Imaging Intelligence, China). The liver window settings were adjusted to

optimize visualization of the liver parenchyma (window width: 200–300 HU; window level: 30–70 HU). The volume of interest (VOI) segmentation included the entire liver parenchyma on CT images, excluding visible benign lesions (e.g., cysts, hemangiomas, and calcifications) and the major veins and branches of the liver. Segmentation was manually adjusted by a junior radiologist (Y.F., with 4 years of experience) using 3D Slicer version 4.10 (www.slicer.org), delineating the liver layer-by-layer while avoiding the liver edge (to prevent partial volume effects), All segmentation masks were reviewed and confirmed by a senior radiologist (T.T., with over 20 years of experience), and any discrepancies were resolved through consensus discussion. Additionally, another senior radiologist (Y.C., with over 20 years of experience) independently performed tumor segmentation and whole-liver segmentation on 40 randomly selected lesions to assess intra-observer reproducibility.

**Appendix E5. Details of radiomics feature extraction**

In our study, 1046 and 1046 radiomic features from T2WI and DWI were calculated based on the segmented primary tumor. Additionally, a set of 1106 radiomic features was computed from the segmented pre-metastatic liver CT images. Since the MRI images were collected from multiple centers, a series of image standardization techniques were applied to process the T2WI and DWI images. The intensity of the T2WI image was normalized using the z-score method, centering it at the mean with a scale of 100.Next, we used a cubic B-spline image interpolation algorithm to resample the T2WI and DWI images to resolutions of [1 × 1 × 1 $mm^3$] and [1.5 × 1.5 × 1.5 $mm^3$], respectively. The grey level of T2WI images was quantized to 5 grey levels. The grey level of the DWI images was quantized to 15 grey levels. After liver segmentation, the 3D CT images were resampled to a new resolution [1 × 1 × 1$mm^3$] using a cubic B-spline interpolation algorithm.

The following 4 types of image features were extracted in this study[3]:

**(1) First-order statistical features**

First-order statistics describe the distribution of voxel intensities within the image region defined by the mask through commonly used and basic metrics.

**(2) Shape-based 3D features**

In this group of features, we included descriptors of the three-dimensional size and shape of the region of interest (ROI). These features are independent of the grey-level intensity distribution in the ROI and are therefore only calculated on the nonderived image and mask.

**(3) Statistics-based textural features**

Statistics-based textural features can reflect the homogeneity phenomenon of the images and the arrangement of the properties that change slowly or periodically on the body surface. The textural features extracted in our study included five types of matrix features, including grey-level co-occurrence matrix (GLCM) features, grey-level run length matrix (GLRLM) features, grey-level size zone matrix (GLSZM) features, neighbouring grey tone difference matrix (NGTDM) features, and grey-level dependence matrix (GLDM) features. Determining the texture matrix representations requires the voxel intensity values within the volume of interest (VOI) to be discretized. Voxel intensities were therefore resampled into equally spaced bins using a bin width of 5 grey levels. This discretization step not only reduces the image noise but also normalizes the intensities across all patients, allowing for a direct comparison of all the calculated textural features between patients.

A GLCM classifier is used to describe the distance and angle of each pixel and calculate the correlation between two grey levels with certain directions and distances. The GLCM can reflect integrated information regarding the direction, interval, amplitude, and frequency of images. For GLRLM, the run length metrics quantify the grey-level runs in an image. A grey-level run is defined as the length of the number of pixels and of consecutive pixels that have the same grey-level value. The GLSZM describes the amount of homogeneous connected areas within the tumour volume of a certain size and intensity, thus reflecting tumour heterogeneity at the regional scale.

### (4) Wavelet and LoG filtration features

The wavelet feature and LoG feature were calculated by filtering the original T2WI, DWI and CT image with a wavelet filter and LoG filter. An LoG spatial bandpass filter was used to derive image features at different spatial scales by tuning the filter parameters to 1.0, 3.0 and 5.0.

Wavelet transformation effectively decouples textural information by decomposing the original image into low- and high-frequency images. A discrete, one-level and undecimated three-dimensional wavelet transformation was applied to each MR image, which decomposed the original image into 8 decompositions. Considering L and H to be low-pass and high-pass functions, respectively, X is the decomposing image, and the wavelet decompositions of X are labelled XLLL, XLLH, XLHL, XLHH, XHLL, XHLH, XHHL, and XHHH. Then, eight new images that are decomposed in three directions (x, y, z) could be obtained. Since the applied wavelet decomposition was undecimated, the size of each decomposition was equal to that of the original image, and each decomposition was shift invariant. Thus, the original tumour delineation of the tumour volume could be applied directly to the decompositions after wavelet transformation.

## Reference

[1] Benson AB, Venook AP, Al-Hawary MM et al (2022) Rectal Cancer, Version 2.2022, NCCN Clinical Practice Guidelines in Oncology. J Natl Compr Canc Netw 20:1139-1167 Doi:10.6004/jnccn.2022.0051

[2] Van Cutsem E, Cervantes A, Nordlinger B, Arnold D (2014) Metastatic colorectal cancer: ESMO Clinical Practice Guidelines for diagnosis, treatment and follow-up. Ann Oncol 25 Suppl 3:iii1-9 Doi:10.1093/annonc/mdu260

[3] van Griethuysen JJM, Fedorov A, Parmar C et al (2017) Computational Radiomics System to Decode the Radiographic Phenotype. Cancer Res 77:e104-e107 Doi:10.1158/0008-5472.Can-17-0339

## Figure S1

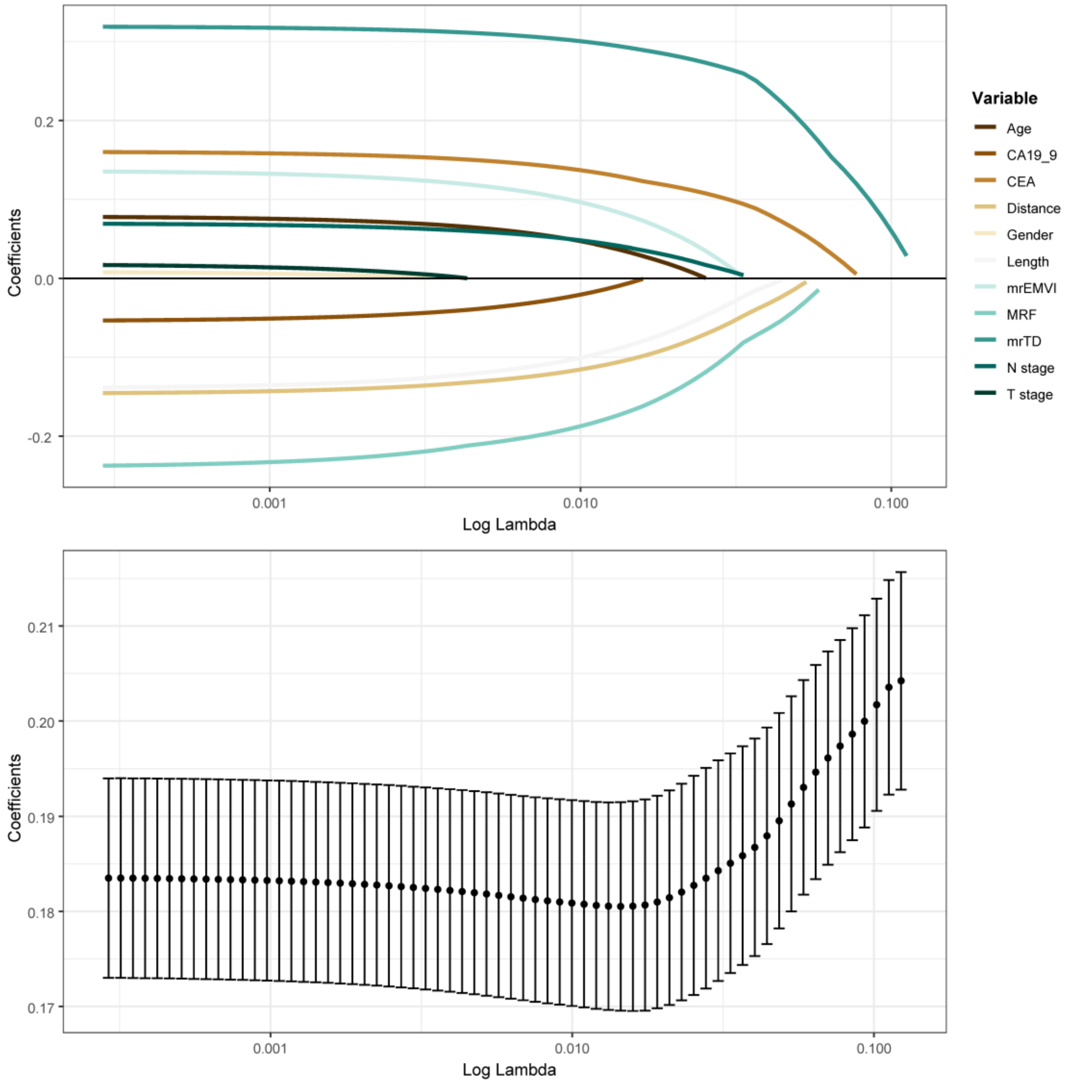


**Figure S1.** Predictor selection performed using LASSO regression analysis for 3-year liver metastasis prediction. *Abbreviations: CA19_9: carbohydrate antigen 19_9; CEA: carcinoembryonic antigen; EMVI, extramural venous invasion; MRF, mesorectal fascia; TD, tumor deposits.*

## Figure S2

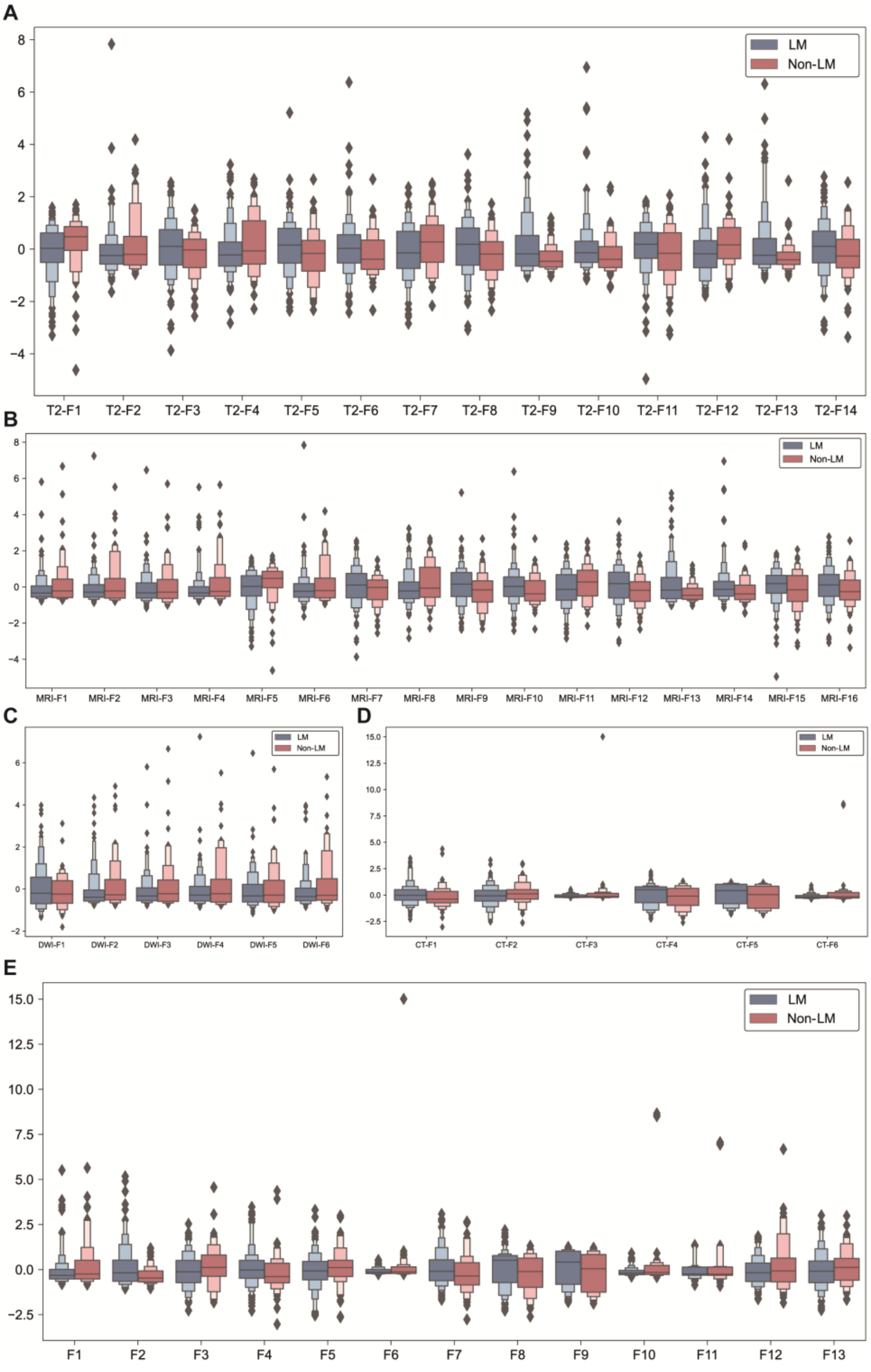


**Figure S2.** The boxplots of the selected radiomic features in the T2WI model, DWI model, the liver CT mdel, the rectal MRI (T2WI+DWI) model and the fusion (T2+DWI+CT) model.

**Figure S3.**

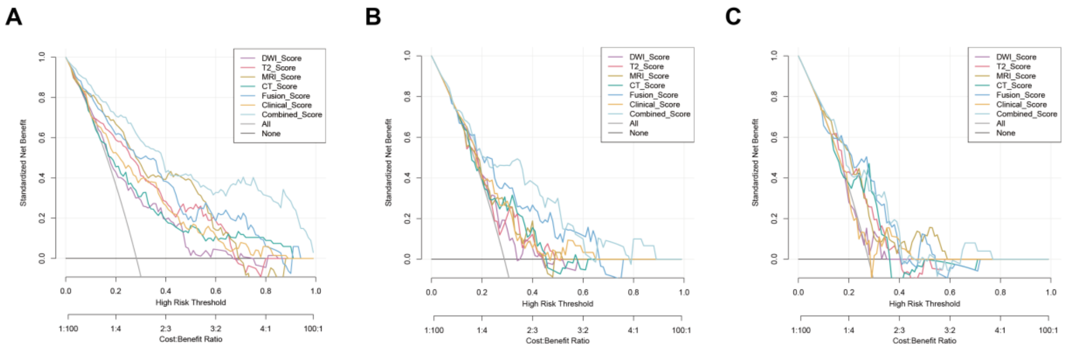


**Figure S3.** The decision curve analysis (DCA) curves of all models.

**Figure S4**

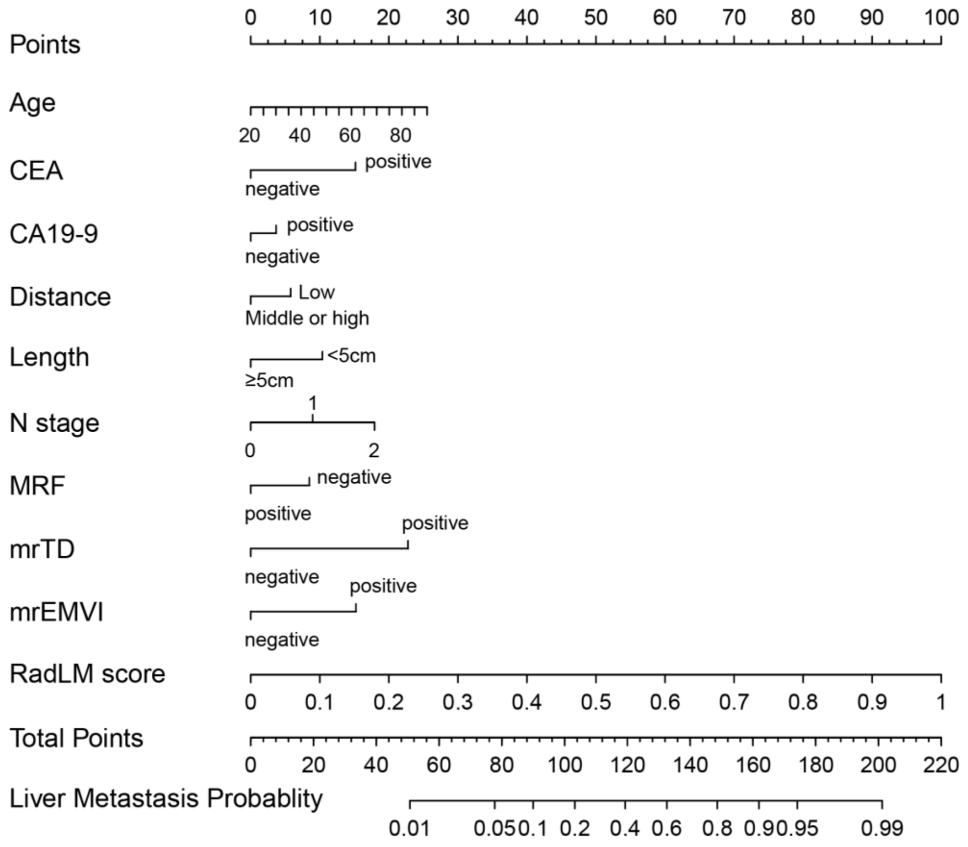


**Figure S4.** Nomogram of the combined predictive model.

**Figure S5.**

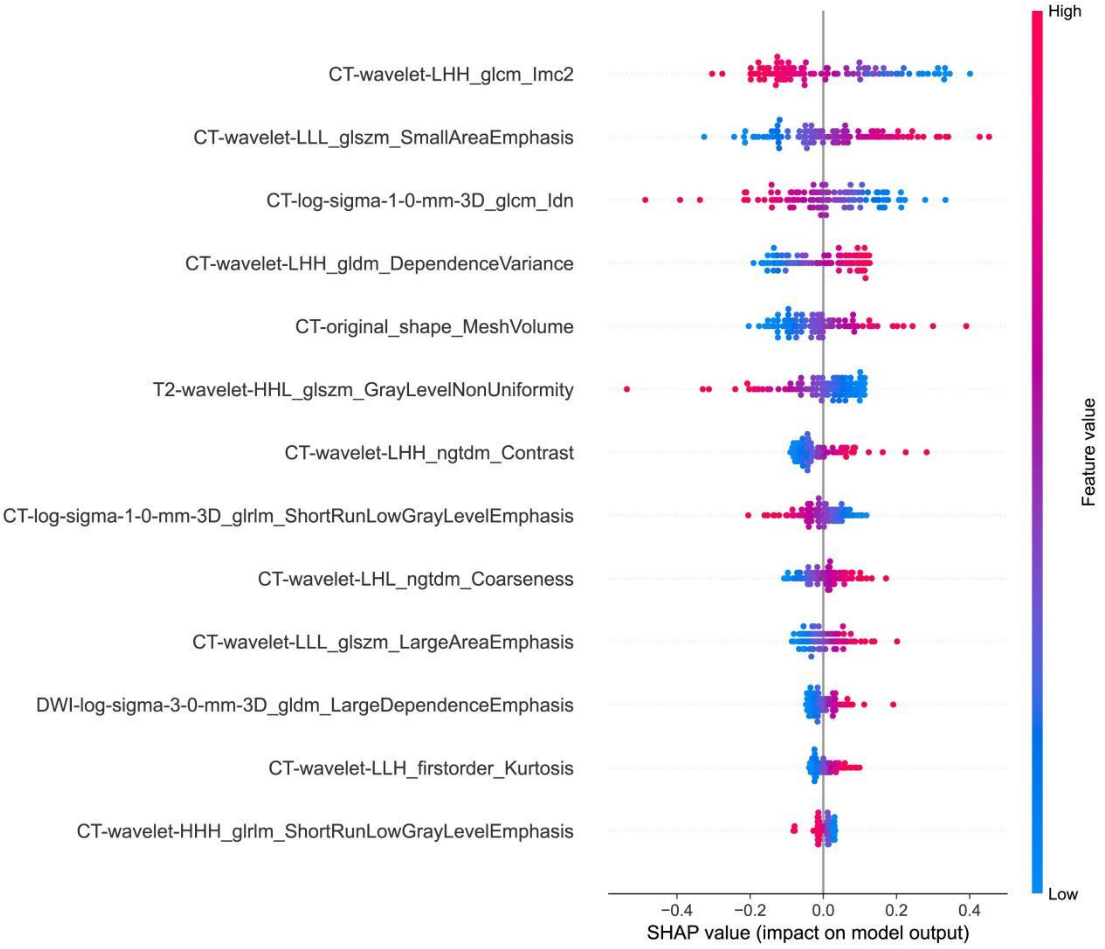


**Figure S5.** SHAP summary plots for the fusion radiomics model, highlighting feature relevance and their contributions to predictive performance.

**Table S1. The detailed MRI protocol parameters at two centers**

| Hospital | Scanner | Sequence Type | TR/TE (ms) | Flip Angle | FOV ($cm^2$) | Slice Thickness (mm) | Slice Gap (mm) | Matrix | b Value ($s/mm^2$) |
|---|---|---|---|---|---|---|---|---|---|
| Fudan University Shanghai Cancer Center | Siemens 3.0T (MAGNETOM skyra) | T2WI | 1500/100 | 135° | 23 x 23 | 1.5 | 0 | 291 x 320 | - |
| | | DWI | 4900/60 | 135° | 18 x 20 | 5.5 | 5.5 | 140 x 120 | 0, 800 |
| | GE 3.0T ( Signa HDxt) | T2WI | 3840/112 | 90° | 25 x 25 | 3 | 4 | 384 x 224 | - |
| | | DWI | 4000/80 | 90° | 18 x 18 | 5.5 | 5.5 | 120×90 | 0, 800 |
| Shanxi Province Cancer Hospital | Philips 3.0T(Achieva) | T2WI | 3000/80 | 90° | 18 x 18 | 3 | 3.3 | 300 x 223 | - |
| | | DWI | 2750/53.4 | 90° | 37.5 x 37.5 | 5 | 5.5 | 124 x 187 | 0, 800 |

Abbreviations: TR, repetition time; TE, echo time; FOV, field of view; T2WI, T2 weighted imaging; DWI, diffusion weighted imaging.

**Table S2: The detailed CT protocol parameters at two centers**

| Item | Details |
|---|---|
| **CT Manufacturer and Model** | Philips (Brilliance 64 ), Siemens (Sensation 40, SOMATOM Definition AS+), GE Medical Systems(Discovery CT750 HD) and TOSHIBA (Aquilion ONE) |
| **Tube Voltage** | 100-120 keV |
| **Tube Current** | 50–400 mAs |
| **Field of View** | 350 × 350–500 × 500 mm |
| **Slice Thickness** | 1.25–5 mm |
| **Contrast Material Bolus** | 1.3–1.5 ml/kg body weight, injected intravenously at a flow rate of 3.0–4.0 ml/s |
| **Arterial Phase Delay** | 20–25 s delay after enhancement of the descending aorta to 100 HU (measured with bolus-tracking technique) |
| **Portal Venous Phase Timing** | 60–75 s after injection of contrast material |

**Table S3. The selected radiomics features**

| | Abbr | Sequences | Features |
|---|---|---|---|
| **T2 radiomics model** | F1 | T2 | original_glcm_Imc1 |
| | F2 | T2 | log-sigma-1-0-mm-3D_firstorder_Kurtosis |
| | F3 | T2 | log-sigma-2-0-mm-3D_glszm_ZoneEntropy |
| | F4 | T2 | wavelet-LLH_glszm_SizeZoneNonUniformityNormalized |
| | F5 | T2 | wavelet-LHH_glrlm_RunVariance |
| | F6 | T2 | wavelet-HLL_firstorder_MeanAbsoluteDeviation |
| | F7 | T2 | wavelet-HLL_glszm_GrayLevelNonUniformityNormalized |
| | F8 | T2 | wavelet-HLL_glszm_ZoneEntropy |
| | F9 | T2 | wavelet-HHL_glszm_GrayLevelNonUniformity |
| | F10 | T2 | wavelet-LLL_firstorder_Maximum |
| | F11 | T2 | wavelet-LLL_glcm_Correlation |
| | F12 | T2 | wavelet-LLL_glcm_MaximumProbability |
| | F13 | T2 | wavelet-LLL_glszm_GrayLevelNonUniformity |
| | F14 | T2 | wavelet-LLL_gldm_DependenceEntropy |
| **DWI radiomics model** | F1 | DWI | DWI-F1 = DWI-original_shape_Maximum2DDiameterSlice |
| | F2 | DWI | DWI-F2 = DWI-log-sigma-1-0-mm-3D_gldm_DependenceVariance |
| | F3 | DWI | DWI-F3 = DWI-log-sigma-2-0-mm-3D_glcm_Autocorrelation |
| | F4 | DWI | DWI-F4 = DWI-log-sigma-3-0-mm-3D_glcm_Autocorrelation |
| | F5 | DWI | DWI-F5 = DWI-log-sigma-3-0-mm-3D_glszm_GrayLevelVariance |
| | F6 | DWI | DWI-F6 = DWI-log-sigma-3-0-mm-3D_gldm_DependenceVariance |
| **MRI (T2+DWI) radiomics model** | F1 | DWI | log-sigma-2-0-mm-3D_glcm_Autocorrelation |
| | F2 | DWI | log-sigma-3-0-mm-3D_glcm_Autocorrelation |
| | F3 | DWI | log-sigma-3-0-mm-3D_glszm_GrayLevelVariance |
| | F4 | DWI | log-sigma-3-0-mm-3D_gldm_LargeDependenceEmphasis |
| | F5 | T2 | original_glcm_Imc1 |
| | F6 | T2 | log-sigma-1-0-mm-3D_firstorder_Kurtosis |
| | F7 | T2 | log-sigma-2-0-mm-3D_glszm_ZoneEntropy |
| | F8 | T2 | wavelet-LLH_glszm_SizeZoneNonUniformityNormalized |
| | F9 | T2 | wavelet-LHH_glrlm_RunVariance |
| | F10 | T2 | wavelet-HLL_firstorder_MeanAbsoluteDeviation |
| | F11 | T2 | wavelet-HLL_glszm_GrayLevelNonUniformityNormalized |

| | | | |
|---|---|---|---|
| | F12 | T2 | wavelet-HLL_glszm_ZoneEntropy |
| | F13 | T2 | wavelet-HHL_glszm_GrayLevelNonUniformity |
| | F14 | T2 | wavelet-LLL_firstorder_Maximum |
| | F15 | T2 | wavelet-HHL_glszm_GrayLevelNonUniformity |
| | F16 | T2 | wavelet-LLL_gldm_DependenceEntropy |
| **CT radiomics model** | F1 | CT | log-sigma-1-0-mm-3D_glcm_Idn |
| | F2 | CT | log-sigma-1-0-mm-3D_glrlm_ShortRunLowGrayLevelEmphasis |
| | F3 | CT | wavelet-LLH_firstorder_Kurtosis |
| | F4 | CT | wavelet-LHH_glcm_Imc2 |
| | F5 | CT | wavelet-LHH_gldm_DependenceVariance |
| | F6 | CT | wavelet-LHH_ngtdm_Contrast |
| **Fusion (T2+DWI+CT) radiomics model** | F1 | DWI | log-sigma-3-0-mm-3D_gldm_LargeDependenceEmphasis |
| | F2 | T2 | wavelet-HHL_glszm_GrayLevelNonUniformity |
| | F3 | CT | original_shape_MeshVolume |
| | F4 | CT | log-sigma-1-0-mm-3D_glcm_Idn |
| | F5 | CT | log-sigma-1-0-mm-3D_glrlm_ShortRunLowGrayLevelEmphasis |
| | F6 | CT | wavelet-LLH_firstorder_Kurtosis |
| | F7 | CT | wavelet-LHL_ngtdm_Coarseness |
| | F8 | CT | wavelet-LHH_glcm_Imc2 |
| | F9 | CT | wavelet-LHH_gldm_DependenceVariance |
| | F10 | CT | wavelet-LHH_ngtdm_Contrast |
| | F11 | CT | wavelet-HHH_glrlm_ShortRunLowGrayLevelEmphasis |
| | F12 | CT | wavelet-LLL_glszm_LargeAreaEmphasis |
| | F13 | CT | wavelet-LLL_glszm_SmallAreaEmphasis |

## Table S4. Delong Tests between AUCs of Different Models

| Models | Training cohort | | Internal validition cohort | | External validition cohort | |
|---|---|---|---|---|---|---|
| | Z score | P value | Z score | P value | Z score | P value |
| **T2 vs DWI** | 1.804 | 0.071 | 0.063 | 0.95 | 1.098 | 0.272 |
| **T2 vs MRI** | -1.77 | 0.077 | -0.534 | 0.593 | -0.677 | 0.498 |
| **T2 vs CT** | 1.418 | 0.156 | -0.368 | 0.713 | -0.247 | 0.805 |
| **T2 vs Fusion** | -1.237 | 0.216 | -1.64 | 0.101 | -0.695 | 0.487 |
| **T2 vs Clinical** | 0.51 | 0.61 | -0.578 | 0.563 | 1.416 | 0.157 |
| **T2 vs Combined** | -2.802 | 0.005 | -2.307 | 0.021 | -0.806 | 0.42 |
| **DWI vs MRI** | -3.226 | <0.001 | -0.355 | 0.723 | -1.243 | 0.214 |
| **DWI vs CT** | -0.225 | 0.822 | -0.427 | 0.67 | -1.17 | 0.242 |
| **DWI vs Fusion** | -2.96 | 0.003 | -1.522 | 0.128 | -1.53 | 0.126 |
| **DWI vs Clinical** | -1.176 | 0.24 | -0.616 | 0.538 | 0.198 | 0.843 |
| **DWI vs Combined** | -4.64 | <0.001 | -2.039 | 0.041 | -1.715 | 0.086 |
| **MRI vs CT** | 2.423 | 0.015 | -0.156 | 0.876 | 0.032 | 0.974 |
| **MRI vs Fusion** | -0.196 | 0.845 | -1.256 | 0.209 | -0.377 | 0.706 |
| **MRI vs Clinical** | 1.434 | 0.151 | -0.349 | 0.727 | 1.579 | 0.114 |
| **MRI vs Combined** | -1.907 | 0.046 | -1.942 | 0.047 | -0.443 | 0.658 |
| **CT vs Fusion** | -4.006 | <0.001 | -1.396 | 0.163 | -0.575 | 0.566 |
| **CT vs Clinical** | -0.89 | 0.374 | -0.169 | 0.866 | 1.359 | 0.174 |
| **CT vs Combined** | -5.292 | <0.001 | -2.199 | 0.028 | -0.48 | 0.631 |
| **Fusion vs Clinical** | 1.656 | 0.098 | 0.894 | 0.371 | 1.697 | 0.09 |
| **Fusion vs Combined** | -2.818 | 0.005 | -1.191 | 0.234 | -0.071 | 0.943 |
| **Clinical vs Combined** | -4.672 | <0.001 | -1.997 | 0.046 | -2.788 | 0.005 |

## Table S5. Subgroup analysis of Cox regression for LM prediction in patients undergoing surgery (Center A).

| Variables | Classification | Numbers (proportion) | HR (univariable) | HR (multivariable) |
|---|---|---|---|---|
| **Age (y)** | <60 | 135 (49.8%) | | |
| | ≥ 60 | 136 (50.2%) | 1.04 (0.78-1.40, p= 0.783 | |
| **Gender** | Female | 86 (31.7%) | | |
| | Male | 185 (68.3%) | 0.85 (0.62-1.16, p= 0.315) | |
| **CEA (ng/mL)** | <5 | 131 (48.3%) | | |
| | ≥5 | 140 (51.7%) | 0.97 (0.72-1.31, p= 0.853) | |
| **CA19_9 (μg/mL)** | <27 | 199 (73.4%) | | |
| | ≥27 | 72 (26.6%) | 1.20 (0.85-1.69, p= 0.291) | |
| **MRF** | Negative | 117 (43.2%) | | |
| | Positive | 154 (56.8%) | 2.19 (1.62-2.97, p< 0.001) | 1.60 (1.11-2.30, p= 0.011) |
| **Distance (cm)** | Low | 151 (55.7%) | | |
| | Middle or high | 120 (44.3%) | 0.99 (0.73-1.32, p= 0.920) | |
| **Length (cm)** | <5 | 124 (45.8%) | | |
| | ≥5 | 147 (54.2%) | 1.27 (0.94-1.71, p= 0.117) | |
| **mrTD** | Negative | 225 (83.0%) | | |
| | Positive | 46 (17.0%) | 1.64 (1.03-2.61, p= 0.039) | 1.12 (0.69-1.82, p= 0.657) |
| **mrEMVI** | Negative | 130 (48.0%) | | |
| | Positive | 141 (52.0%) | 2.32 (1.70-3.17, p< 0.001) | 1.72 (1.20-2.46, p= 0.003) |
| **Clinical stage** | Ⅰ | 8 (3.0%) | | |
| | II | 48 (17.7%) | 1.36 (0.57-3.23, p= 0.482) | |
| | III | 215 (79.3%) | 1.72 (0.76-3.92, p= 0.195) | |
| **RadLM score** | Low-risk | 165 (60.9%) | | |
| | High-risk | 106 (39.1%) | 3.52 (2.01-6.17, p< 0.001) | 2.31 (1.30-4.10, p= 0.004) |
| **Postoperative_Chemotherapy** | No | 77 (28.4%) | | |
| | Yes | 194 (71.6%) | 1.12 (0.82-1.54, p= 0.467) | |

Abbreviations: CA19_9: carbohydrate antigen 19_9; CEA: carcinogenicity antigen; EMVI, extramural venous invasion; MRF, mesorectal fascia; TD, tumor deposits; HR: hazard ratio
